# High‐throughput CRISPR screen of GWAS risk loci in human microglia reveals novel risk genes for Alzheimer's disease and multiple sclerosis

**DOI:** 10.1002/alz70855_105086

**Published:** 2025-12-24

**Authors:** Michael D Gallagher, Wenjuan Du, Moritz List, Katheryn EA Hazel, Xochitl Luna, Zeynep Aydin, Yiran Cheng, Bingbing Yuan, Keya Viswanathan, Heather M Keys, George W Bell, Richard A Young, Rudolf Jaenisch, Olivia Corradin

**Affiliations:** ^1^ Whitehead Institute, Cambridge, MA, USA; ^2^ Massachusetts Institute of Technology, Cambridge, MA, USA

## Abstract

**Background:**

Alzheimer's (AD) and other neurological diseases (NDs) cause substantial health‐related and economic burdens, but progress toward preventative or ameliorative treatments has been limited. Genome‐wide association studies (GWAS) have identified hundreds of risk loci containing single nucleotide polymorphisms (SNPs) that alter ND risk, but >90% of these SNPs are in noncoding regions, which are cell type‐specific and difficult to study. To address this limitation, we have combined iPSC‐based cell models and functional genomics to pinpoint the causal genes at 10 loci associated with risk for AD and multiple sclerosis (MS).

**Methods:**

We generated neural progenitors, excitatory neurons, astrocytes and microglia from iPSCs and performed RNA‐seq, H3K27ac ChIP‐seq and Promoter Capture Hi‐C. We compared these data to those from uncultured primary (ex vivo) human brain cells to assess their physiological relevance, and determined global patterns of ND risk SNP enrichments in cell type‐specific enhancers. We transduced iPSC‐derived microglia‐like cells (iMGLs) with a lentiviral sgRNA library targeting AD and MS risk SNPs and performed scRNA‐seq to identify altered genes and cellular pathways.

**Results:**

iPSC‐derived cell types displayed gene signatures and enhancer patterns consistent with their cell identities, with iMGLs displaying the strongest similarity to primary cells. Neuronal and glial cells from both iPSC and primary brain sources show consistent enrichment of ND risk SNPs in cell type‐specific enhancers, with microglia showing enrichment of AD and MS SNPs. Enhancer/promoter interactions identified in iMGLs and primary microglia nominated potential target genes of these SNPs, and these predictions were tested by CRISPRi single cell screening. We identified multiple novel AD and MS risk genes, including the transcription factor MAF, which is regulated by both AD and MS risk SNPs, and ELMO1, which we characterize as a novel MS risk gene involved in microglial homeostasis.

**Conclusions:**

The combination of iPSC‐based cell models and functional genomics allowed for systematic perturbation of AD and MS risk SNPs, revealing previously uncharacterized risk genes linking neuroimmune pathways to disease pathophysiology. Ongoing studies are exploring the role of altered expression levels of MAF, ELMO1 and other screen hits in microglial phenotypes, including effects on neurons and astrocytes in co‐culture and organoid systems.

